# A Polydnavirus ANK Protein Acts as Virulence Factor by Disrupting the Function of Prothoracic Gland Steroidogenic Cells

**DOI:** 10.1371/journal.pone.0095104

**Published:** 2014-04-17

**Authors:** Luca Valzania, Patrizia Romani, Ling Tian, Sheng Li, Valeria Cavaliere, Francesco Pennacchio, Giuseppe Gargiulo

**Affiliations:** 1 Dipartimento di Farmacia e Biotecnologie, Università di Bologna, Bologna, Italy; 2 Key Laboratory of Insect Developmental and Evolutionary Biology, Institute of Plant Physiology and Ecology, Shanghai, China; 3 Dipartimento di Agraria – Laboratorio di Entomologia “E. Tremblay”, Università di Napoli ‘Federico II’, Portici (NA), Italy; National Cancer Institute, United States of America

## Abstract

Polydnaviruses are obligate symbionts integrated as proviruses in the genome of some ichneumonoid wasps that parasitize lepidopteran larvae. Polydnavirus free viral particles, which are injected into the host at oviposition, express virulence factors that impair immunity and development. To date, most studies have focused on the molecular mechanisms underpinning immunosuppression, whereas how viral genes disrupt the endocrine balance remains largely uninvestigated. Using *Drosophila* as a model system, the present report analyzes the function of a member of the *ankyrin* gene family of the bracovirus associated with *Toxoneuron nigriceps*, a larval parasitoid of the noctuid moth *Heliothis virescens*. We found that the *TnBVank1* expression in the *Drosophila* prothoracic gland blocks the larval-pupal molt. This phenotype can be rescued by feeding the larvae with 20-hydroxyecdysone. The localization of the *Tn*BVANK1 is restricted to the cytoplasm where it interacts with Hrs and Alix marked endosomes. Collectively, our data demonstrate that the *Tn*BVANK1 protein acts as a virulence factor that causes the disruption of ecdysone biosynthesis and developmental arrest by impairing the vesicular traffic of ecdysteroid precursors in the prothoracic gland steroidogenic cells.

## Introduction

Parasitic wasps represent the largest group of parasitoid insects which attack and parasitize a number of insect species, exploiting different developmental stages [1]. These parasitic insects have a peculiar injection device, the ovipositor, which is used to deliver the egg along with host regulation factors that primarily disrupt the host immune reaction and endocrine balance to create a suitable environment for the development of their progeny [2], [3]. These host regulation factors include viruses of the Polydnaviridae family, obligate symbionts of ichneumonid and braconid wasps attacking larval stages of lepidopteran hosts, and respectively classified in the genera *Ichnovirus* (IV) and *Bracovirus* (BV) [4].

Polydnaviruses (PDVs) [5], [6] are integrated as proviruses in the genome of parasitoid wasps and their transmission to offspring is strictly vertical, through the germline. The genome encapsidated in the viral particles is made of multiple circular dsDNA segments, which have an aggregate size ranging between 190 and 600 kb. PDVs only replicate in the epithelial cells of the calyx, a specific region of the ovary, where they accumulate to a high density to be injected at oviposition along with the venom and the egg. Free PDV particles infect the host tissues without undergoing replication, and express virulence factors that alter host physiology in ways essential for offspring survival [5].

Evolutionary convergence of independent host-virus associations has favored the selection of gene families shared by both IV and BV [7]–[9]. For example, protein tyrosine phosphatases (PTP) and ankyrin motif proteins (ANK) are widely distributed in many PDVs and expressed to different degrees in virtually all host tissues analyzed so far, indicating that they play a key role in successful parasitism [10].

We know more about the functional bases underlying the immune disguise in parasitized hosts than we do about how the host developmental alteration is induced [3], [11]. This is due to the complexity of the developmental mechanisms and to the concurrent action of various virulence factors, which often have redundant and overlapping effects on the regulating gene networks [2], [3], [12].

One of the best characterized developmental syndromes has been described in the host-parasitoid association *Heliothis virescens-Toxoneuron nigriceps* (Lepidoptera, Noctuidae - Hymenoptera, Braconidae) [13]. Briefly, in this experimental model the last instar larvae fail to pupate and show a higher nutritional suitability for parasitoid larvae. The developmental arrest is partly due to a marked depression of ecdysone (E) biosynthesis by the prothoracic gland (PG), induced by the infection of the bracovirus associated with *T. nigriceps* (*Tn*BV). The inhibition of E biosynthesis is further reinforced by the conversion of the very low amounts of 20-hydroxyecdysone (20E) produced to inactive polar metabolites, a transformation mediated by teratocytes, special cells deriving from the parasitoid's embryonic membrane.

The active transcription of *Tn*BV genes in the PG of parasitized tobacco budworm larvae is required to disrupt their ecdysteroid biosynthesis, which remains very low and fails to increase in response to prothoracicotropic hormone (PTTH) stimulation [13], [14]. Unraveling the functional role of a specific virulence factor at molecular level is not easy when the natural host is used for these studies, due to the limited availability of genomic information and molecular tools. Therefore we used *Drosophila melanogaster* as an ideal experimental model to study *Tn*BV genes. With this approach, we started the functional characterization of a member of the viral *ankyrin* (*ank*) gene family of *Tn*BV, *TnBVank1* [15], showing that the expression of this gene in *Drosophila* germ cells alters the microtubule network function in the oocyte [16]. In the present study we analyze the effect of *TnBVank1* gene expression during *Drosophila* development. Interestingly, we found that *TnBVank1* expression in the PG cells blocks the transition from larval to pupal stage, mimicking the developmental arrest observed in *H. virescens* larvae parasitized by *T. nigriceps*.

## Materials and Methods

### Fly strains

Stocks were raised on standard cornmeal/yeast/agar medium at 21°C and crosses were made at 25°C unless otherwise stated. *yw^67c23^* was used as the wild-type stock in this study. The *UASp-TnBVank1* strain (genotype: *UASp-TnBVank1/UASp-TnBVank1;UASp-TnBVank1/UASp-TnBVank1; +/+*) was generated in our laboratory [16].

The following stocks were obtained from the Bloomington Stock Center: hairy-Gal4 (#1734: w^*^; P{GawB}h^1J3^), UASp-α-tubulin-GFP (#7374: y^1^ w^*^; P{UASp-GFPS65C-αTub84B}14-6-II), UAS-p35 (#5073: w^*^; P{UAS-p35.H}BH2) and tub-Gal80^ts^ (#7019: w^*^; P{w^+mC^ = tubP-GAL80^ts^}20; TM2/TM6B, Tb).

*phantom-Gal4* and *P0206-Gal4* were a gift from C. Mirth (*phantom-Gal4,UAS-mCD8::GFP/TM6B and P0206-Gal4,UAS-mCD8::GFP*), *august21-Gal4;phantom-Gal4* was kindly provided by M. Jindra (*august21-Gal4/CyO;phantom-Gal4/phantom-Gal4*).

The stocks used for *Gal4* driven expression of *UASp-TnBVank1* referred in Figure S2 and listed in Table S1 are from Bloomington Stock Center.

### Crosses

Females *UASp-TnBVank1* were crossed to males of the different *Gal4* lines. As control, females *yw^67c23^* were crossed to males of the same *Gal4* lines.

For microtubules analysis, females *UASp-TnBVank1* were crossed to males *UASp-α-tubulin-GFP; phantom-Gal4*.

For Gal80^ts^ experiment, females UASp-TnBVank1 were crossed to males tub-Gal80^ts^;phantom-Gal4,UAS-mCD8::GFP/TM6B and females yw^67c23^ were crossed to males tub-Gal80^ts^;phantom-Gal4,UAS-mCD8::GFP/TM6B as control.

To coexpress p35 and *Tn*BVANK1 in PG cells females *UASp-TnBVank1;UASp-TnBVank1;UAS-p35* were crossed to males *phantom-Gal4,UAS-mCD8::GFP/TM6B*.

### Larval length measurements

Five *UASp-TnBVank1/+;UASp-TnBVank1/+;hairy-Gal4/+* larvae at different days after egg deposition (AED) and five control larvae were ice-anesthetized and photographed using a Nikon Eclipse 90i microscope. Images were taken at 4X magnification and the larval length was measured with NIS-Elements Advanced Research 3.10 software.

### 20E titer

Five larvae at different developmental stages were collected and washed with PBS and immediately frozen by liquid nitrogen. Samples were added 200 µl of methanol, homogenized and transferred into 1.5 ml plastic tubes. After 10 minutes centrifugation (12,000 rpm at 4°C) the supernatant was collected into a new tube, the precipitate was re-extracted with 200 µl of methanol and the supernatant was added to the previous one. After 30 minutes on ice, the samples were centrifuged following the same conditions. Samples were dried to remove methanol and then dissolved in the borate buffer. The standard curve was generated according to the standard process of the RIA protocol [17] and then the 20E titer in samples was calculated.

### Rescue experiment

*UASp-TnBVank1/+;UASp-TnBVank1/+;phantom-Gal4,UAS-mCD8::GFP/+* and control larvae were collected at 106 h AED and placed in three groups of ten individuals at 25°C in new tubes supplemented with 20E (Sigma) dissolved in ethanol at 1 mg/ml. Control larvae were fed only with ethanol.

### Prothoracic gland and cellular size measurements

For measurements of PG area and its cellular size, confocal images of 50 PGs taken at 40X magnification were quantified with ImageJ software.

### Statistical analysis

Statistical comparison of mean values was performed by unpaired t-test, using GraphPad Prism 4 software.

### Immunofluorescence microscopy

Larvae were dissected at room temperature in 1xPBS pH 7.5 (PBS) and fixed in 4% formaldehyde for 20 minutes at room temperature. After three washes in PBS, larvae were permeabilized in PBT (PBS pH 7.5+0.3% Triton X-100) for 1 h, washed three times 5 minutes each in PBT and 10 minutes in PBT+2%BSA solution. After that, the larvae were incubated, overnight at 4°C, with primary antibodies diluted in PBT+2%BSA. Larvae were washed three times 10 minutes each in PBT, 10 minutes in PBT+1%BSA solution and incubated 2 hours at room temperature on a rotating wheel with secondary antibodies diluted in PBT+1%BSA. After several washes in PBT, the ring glands were dissected and mounted on microscopy slides in Fluoromount G (Electron Microscopy Sciences). Subsequently samples were analyzed by conventional epifluorescence with a Nikon Eclipse 90i microscope or with TCS SL Leica confocal system. Images were processed using Adobe Photoshop CS4 and Adobe Illustrator CS4.

TRITC-Phalloidin staining was carried out, after incubation with secondary antibodies, by washing larvae three times with PBS and then by incubating larvae for 20 minutes with TRITC-Phalloidin (40 µg/ml in PBS, Sigma).

For Propidium Iodide nuclear counterstaining, the larvae were treated with RNase A (400 µg/ml in PBT, Sigma) overnight at 4°C. After three washes in PBT, the larvae were labeled for 2 hours with Propidium Iodide (10 µg/ml in PBT, Molecular Probes).

The following primary antibodies were used: polyclonal rabbit anti-Dib 1∶200 [18], anti-Cleaved Caspase-3 1∶25 (9661, Cell signaling Technology), anti-Rab7 1∶2000 [19] and anti-Rab11 1∶5000 [19] were detected with DyLight 649-conjugated goat anti-rabbit 1∶500 (Jackson). Polyclonal rabbit anti-*Tn*BVANK1 1∶200 [16] was detected using Cy3- (1∶1000) and DyLight 649- (1∶500) conjugated goat anti-rabbit (Jackson). Monoclonal mouse P1H4 anti-Dynein heavy chain 1∶200 [20], anti-Rab5 1∶25 (610281, BD Biosciences) and anti-Alix 1∶100 [21] were detected with Cy3-conjugated goat anti-mouse 1∶1000 (Jackson). Polyclonal guinea pig anti-Hrs 1∶1000 [22] was detected with DyLight 649-conjugated goat anti-guinea pig 1∶500 (Jackson).

### Terminal deoxynucleotidyl transferase-mediated dUTP Nick End Labeling (TUNEL)

Five days AED larvae were dissected at room temperature in PBS, fixed in 4% formaldehyde for 20 minutes according to a protocol previously described [23]. After TUNEL incubation, anti-Digoxigenin 1∶100 (Roche) was detected with Cy3-conjugated goat anti-mouse 1∶1000 (Jackson).

### Filipin and Oil Red O staining

Ring glands were fixed in 4% formaldehyde for 20 minutes and washed three times in PBS for 5 minutes each. Samples were stained with 50 µg/ml of filipin (Sigma) for 1 h or incubated in an Oil Red O (Sigma) solution at 0.06% for 30 minutes. After incubation tissues were washed twice with PBS before mounting in Fluoromount-G. Samples were analyzed by conventional epifluorescence with a Nikon Eclipse 90i microscope or with a Nikon Eclipse 90i confocal microscope. Images were processed using Adobe Photoshop CS4 and Adobe Illustrator CS4.

### Colocalization analysis

Thresholds of confocal images were set in Adobe Photoshop CS4 to exclude background staining. 509 Hrs positive vesicles were analyzed per *Tn*BVANK1 and Hrs staining. 443 *Tn*BVANK1 positive vesicles were analyzed per *Tn*BVANK1 and Alix staining. 118 Hrs positive vesicles were analyzed per Alix and Hrs staining.

Images were processed with the CDA plugin of ImageJ to obtain Pearson's coefficient (from +1 = complete correlation, to −1 = anti-correlation with 0 = no correlation) [24].

## Results

### Expression of *TnBVank1* in the prothoracic gland induces developmental arrest at third instar larvae

**Figure 1:**
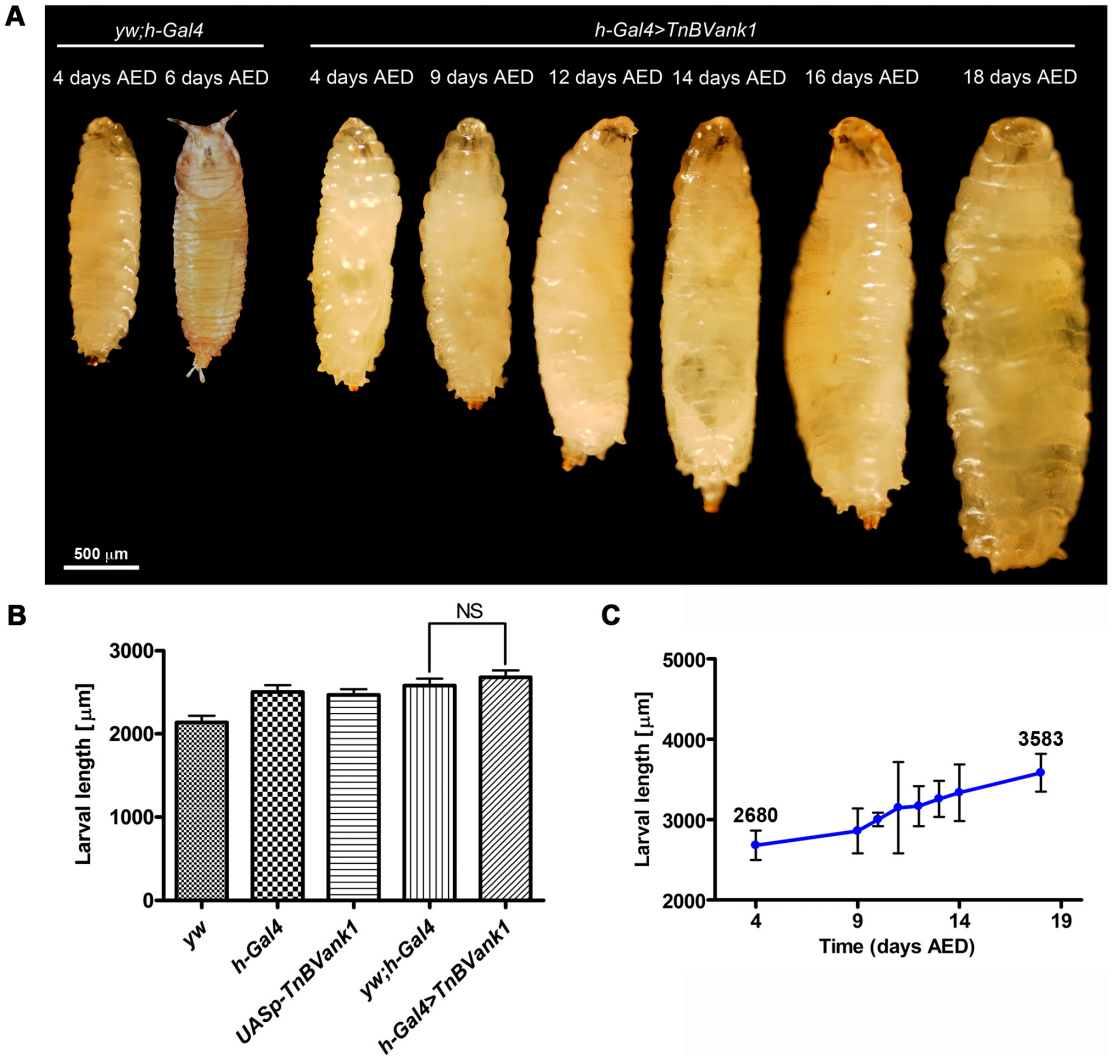
*TnBVank1* expression causes the block of the transition from larval to pupal stage. (A) Light micrographs of *yw;h-Gal4* larva and pupa (control) and *h-Gal4>TnBVank1* larvae at different days AED. The scale bar is 500 µm. (B) Larval length of different genotypes, at 96 h AED. Five larvae of each genotype were analyzed and as control we measured larval length of *yw* and *h-Gal4* and *UASp-TnBVank1* stocks and *yw;h-Gal4*. Graph represents mean ± standard deviation (s.d.); there is no significant (NS) length difference between *h-Gal4>TnBVank1* (2680±83 µm) and *yw;h-Gal4* larvae (2580±82 µm). (C) Larval length of *h-Gal4>TnBVank1* increases during the extended larval life. Five *h-Gal4>TnBVank1* larvae were measured at different days AED; values are the mean ± s.d. of three independent experiments. The mean values of *h-Gal4>TnBVank1* larval length at four and eighteen days AED are shown above the bars.

*TnBVank1* gene expression during *Drosophila* development was targeted with the GAL4/UAS binary system [25]. We used a transgenic *Drosophila* stock carrying two copies of the *TnBVank1* gene under the control of the *UASp* sequences [16]. Expression of this transgene was induced using different *Gal4* drivers. Our first analysis expressed the *TnBVank1* transgene during embryonic and larval development using the *hairy-Gal4* driver (*h-Gal4*) [25]. *TnBVank1* expression did not appear to affect embryonic and larval development but, interestingly, all larvae failed to pupate and died after an extended third instar larval life, which lasted up to three weeks (Figure 1A). By measuring larval size, we found that four days AED the larvae expressing *TnBVank1* did not significantly differ from control *yw;h-Gal4* (n = 5; t = 0.8557; NS) (Figure 1B). Moreover, they continued to feed and significantly increased in size during their prolonged larval life, reaching at eighteen days the maximal length (Figure 1A,C; n = 5; t = 6.765; p<0.0001), while control regularly pupated on day six AED (Figure 1A). Since *h-Gal4* is expressed in various larval tissues, the observed developmental arrest suggested us that *TnBVank1* expression could have reasonably affected the ring gland function, the major site of production and release of developmental hormones.

**Figure 2:**
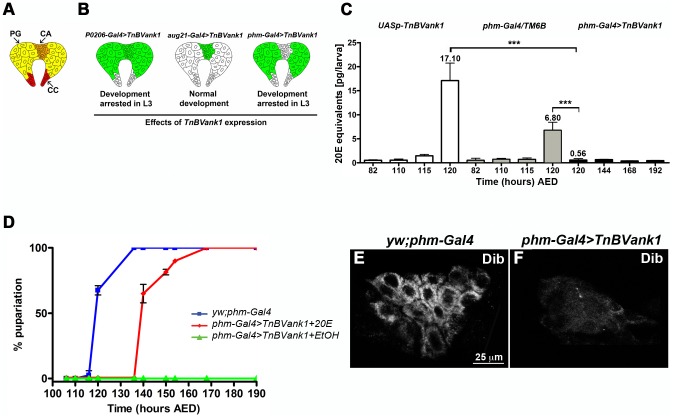
The expression of *TnBVank1* in prothoracic gland affects the E biosynthesis. (A) Ring gland includes the prothoracic gland (PG; yellow), the corpora allata (CA; orange) and the corpora cardiaca (CC; red). (B) The expression of the *TnBVank1* gene is driven in the different ring gland compartments, highlighted in green, by three *Gal4* drivers. *P0206-Gal4>TnBVank1*, expressed in PG and CA, causes the developmental arrest at the last larval stage; *aug21-Gal4>TnBVank1* (CA) does not induce any developmental defects; *phm-Gal4>TnBVank1* (PG) blocks the transition from larval to pupal stage. (C) Total 20E titer in five larvae of *UASp-TnBVank1* stock (white bars), *phm-Gal4/TM6B* (grey bars) and *phm-Gal4>TnBVank1* (black bars), at different time (hours AED). In the control stocks *UASp-TnBVank1* and *phm-Gal4/TM6B*, the 20E peak which induces the pupariation is present at 120 h AED. Instead, this peak is absent in *phm>TnBVank1* larvae at 120 h AED and during the extended larval life. Error bars represent s.d.; *** = p<0.0001 versus controls (*UASp-TnBVank1* and *phm-Gal4/TM6B*). The mean values of total 20E at 120 h AED of different genotype larvae are shown above the bars. (D) Feeding *TnBVank1* larvae with medium supplemented with 20E induces the pupariation (red), while *TnBVank1* larvae fed with medium containing ethanol (EtOH) do not reach the pupal stage (green). Values are the mean ± s.d. of three independent experiments. The *yw;phm-Gal4* larvae serve as background control (blue). Immunostaining with anti-Dib in *yw;phm-Gal4* (E) and *TnBVank1* (F) PG reveals that the expression of Dib is strongly reduced in all *TnBVank1* PGs analyzed. Panels E,F are at the same magnification and the reference scale bar is 25 µm indicated in E.

The *Drosophila* ring gland (Figure 2A) consists of the prothoracic gland (PG), which is composed of steroidogenic cells synthesizing the E, the corpora allata (CA) that produce the juvenile hormone, and the corpora cardiaca (CC), which play a key role in the regulation of metabolic homeostasis [26]. We assessed if the targeted expression of the *TnBVank1* gene using different ring gland *Gal4* drivers (Figure 2B) was able to reproduce the effect observed when the transgene was expressed using the *h-Gal4*. When the *TnBVank1* gene was expressed in both CA and PG cells, using the *P0206-Gal4* driver, all the larvae failed to pupate and showed the same phenotype obtained with *h-Gal4*. Conversely, when the *august21-Gal4* (*aug21-Gal4*) driver specifically targeted the expression of *TnBVank1* in the CA, no effects on developmental timing were observed and regular progeny were obtained. Moreover, we specifically induced expression of the *TnBVank1* gene in the PG using the *phantom-Gal4* (*phm-Gal4*) driver, which is strongly expressed in this gland. None of the larvae pupated and they had an extended larval life as shown using the *P0206-Gal4* driver (Figure 2B). These data indicate *Tn*BVANK1 impairs PG function causing the block of larval-pupal transition.

We specifically expressed *TnBVank1* in several other tissues, using different *Gal4* drivers, and monitored the timing of development and the adult phenotype, which were in all cases not affected (Table S1).

Collectively, our data suggest that the expression of *Tn*BVANK1 has the potential to interfere with the steroid biosynthesis, as further indicated by the targeted expression of this viral ANK protein in PG, which is characterized by developmental arrest of mature larvae and the absence of a systemic injury response [27].

### Ecdysteroid biosynthesis is impaired in *TnBVank1* larvae

To assess whether the developmental arrest induced by *Tn*BVANK1 was due to a reduced level of 20E, we measured the whole body 20E titer in larvae expressing *Tn*BV*ank1* by the *phm-Gal4* driver and in control larvae (Figure 2C). At 110 h AED, wild type third instar larvae enter the wandering stage and, at 25°C, they become white pre-pupae at 120 h, after the surge of a 20E peak [17]. At 120 h AED and during their abnormal extended larval life, the 20E levels measured in *phm-Gal4>TnBVank1* larvae are extremely reduced and significantly lower than that measured both in *UASp-TnBVank1* larvae (n = 5; t = 10.12; p<0.0001) and in *phm-Gal4/TM6B* larvae (n = 5; t = 8.196; p<0.0001).

To further demonstrate that the block of the transition to pupal stage showed by the *phm>TnBVank1* larvae (hereinafter *TnBVank1* larvae) was actually due to a low level of 20E, we carried out a 20E-feeding rescue experiment. Third instar *TnBVank1* larvae were fed with yeast paste containing 20E dissolved in ethanol at 106 h AED, just before the onset of the ecdysteroid peak occurring in the wild-type. As expected, at 120 h AED, 70% of control larvae started to pupate and within the following 20 h all of them reached the pupal stage (n = 30). Pupation of *TnBVank1* larvae fed with 20E followed almost an identical pattern, with 100% pupation (n = 30) attained only 1 day later, but failed to progress to the pharate stage (Figure 2D). Instead, *TnBVank1* larvae treated only with yeast and ethanol persisted as third instar (n = 30). This result confirms that the developmental arrest of *TnBVank1* larvae is due to a reduced level of 20E. However, the rescued pupae failed to develop into adult flies. This may be due to the fact that the large peak of 20E required to trigger metamorphosis is not generated by *TnBVank1* pupae and cannot obviously be supplied with food at this developmental stage.

It has been reported that a positive feedback is required for the transcriptional up-regulation of enzymes acting at late steps in the ecdysone biosynthetic pathway [28]. Therefore we analyzed the expression of Disembodied (Dib), the downstream step enzyme C22 hydroxylase, which appeared strongly reduced in all *TnBVank1* PGs analyzed (n = 60) compared to control (Figure 2E,F). This data is in agreement with low levels of 20E detected in *TnBVank1* larvae.

### *Tn*BVANK1 affects PG morphology

**Figure 3:**
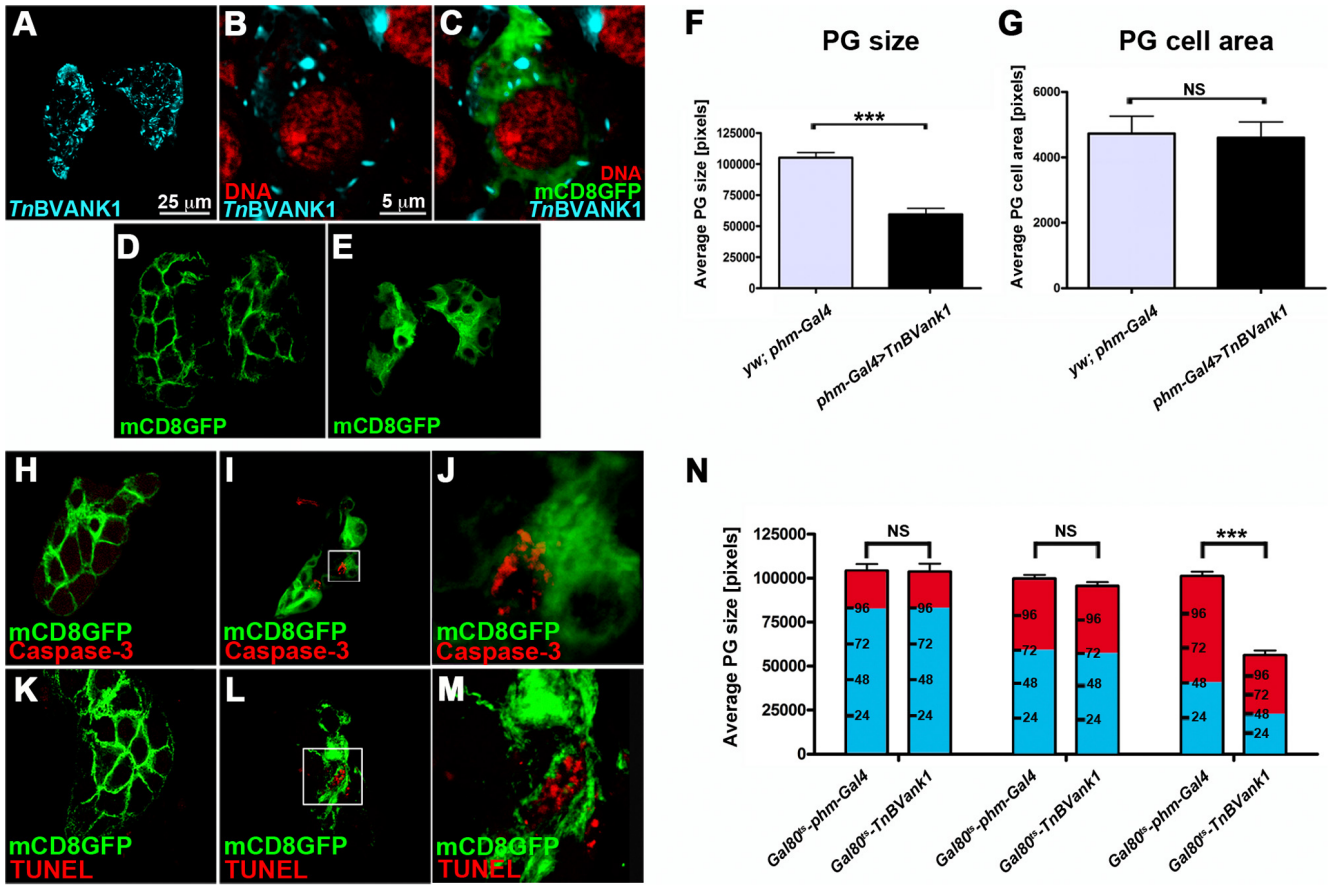
*Tn*BVANK1 distribution in the PG cells and its effects on PG. The immunolocalization of *Tn*BVANK1 in PG cells (marked with mCD8::GFP, green), analyzed with anti-*Tn*BVANK1 (cyan), shows its presence in stroke-shaped particles (A–C), which are distributed only in cytoplasm (nucleus is stained with Propidium Iodide, red) (B,C). (D) At five days AED, the PG of *yw;phm-Gal4* larvae, marked with GFP, is significantly larger (+54%) than the *TnBVank1* PG (E). (F) The graph represents the mean ± s.d.; 50 PGs were analyzed; *** = p<0.0001. (G) Measurement of PG cell area shows no difference between *yw;phm-Gal4* and *TnBVank1*. 50 PGs were analyzed; NS: not significant. (H–J) Immunostaining with anti-Cleaved Caspase-3 (red) or TUNEL labeling (red) in PG cells marked with GFP. In the control *yw;phm-Gal4* no caspase or TUNEL signal is detected (H,K), while in *TnBVank1* PG few cells undergo apoptosis (I,J,L,M). PGs in panels A,D,E,H,I,K,L are at the same magnification and their scale bar is 25 µm and is showed in A. Scale bar in B and C is 5 µm and showed in B. Boxed regions are magnified in J and M and the reference scale bar is in B. (N) Larvae of *yw;tub-Gal80^ts^/+;phm-Gal4/+* (*Gal80^ts^-phm-Gal4*) and *UASp-TnBVank1;UASp-TnBVank1/tub-Gal80^ts^;phm-Gal4/+* (*Gal80^ts^-TnBVank1)* were raised at 21°C (cyan) for different time intervals, then shifted at 31°C (red) and dissected at 120 h AED. PG size from larvae incubated at 21°C until 96 h AED or until 72 h AED shows no significant (NS) differences from control larvae. PG size is strongly reduced in *Gal80^ts^-TnBVank1* larvae incubated at 21°C until 48 h AED compared to PG from control larvae (*** = p<0.0001). Graph represents mean ± s.d.; 10 PGs were analyzed for each experiment.

Using a polyclonal antibody raised against two synthetic peptides of *Tn*BVANK1 [16], we detected the distribution of *Tn*BVANK1 protein in *TnBVank1* PGs of five days AED larvae. As shown in Figure 3A–C, the protein was strongly expressed and present only in the cytoplasm of PG cells, confined to stroke-shaped particles. We next analyzed the *TnBVank1* PG gross morphology. To visualize the PG we used *phm-Gal4,UAS-mCD8::GFP* stock. PGs from control larvae (Figure 3D) were significantly larger (n = 50; t = 50.41; p<0.0001) (Figure 3F) than *TnBVank1* PGs (Figure 3E). In addition, the *TnBVank1* PG cells showed a cytoplasmic rather than the expected membrane distribution of mCD8::GFP (Figure 3C,E) [29]. Measurements of the PG cell area did not show any reduction induced by *TnBVank1* expression (n = 50; t = 1.262; NS) (Figure 3G). Therefore, the observed size difference of PG can be attributed to a reduction of the cell number. We then assayed if apoptosis occurs, using Cleaved Caspase-3 antibody [30] and TUNEL labeling [31]. The Caspase-3 activity (Figure 3I,J; n = 60) and TUNEL positive staining (Figure 3L,M; n = 60) found in a few cells of *TnBVank1* PGs suggested that the occurrence of cell death during development can partly account for this difference, which could be related to the developmental arrest induced by *Tn*BVANK1. However, the possibility that this protein can also disrupt PG activity cannot be ruled out. Therefore, to assess the relative contribution of these two effects, not mutually exclusive, we expressed *TnBVank1* in PG cells at different time points during larval life, using a temperature sensitive form of the Gal4 repressor Gal80, Gal80^ts^ [32], that allows to regulate the *phm-Gal4* activity. *TnBVank1* and control larvae were initially raised at 21°C, and then shifted to the restrictive temperature (31°C) at specific time points (96 h, 72 h and 48 h AED) to promote Gal4 activity. The temperature shift did not affect the proper development of the control larvae, which pupate normally. Conversely, the larvae expressing *TnBVank1* failed to pupate, increased their size and survived for an extended period.

For each time point we also analyzed the PG size at 120 h AED (Figure 3N). When the *TnBVank1* expression was triggered at 96 h or 72 h, the PGs size was not significantly different from controls (respectively n = 10; t = 0.07636; NS and n = 10; t = 1.336; NS). While the earlier induction of the transgene expression, at 48 h AED, strongly affected the PG size, which appeared significantly reduced (n = 10; t = 11.68; p<0.0001). In addition, we examined whether by inhibiting apoptosis with ectopic expression of p35 [33] it would be possible to rescue the phenotype produced by the expression of *TnBVank1* in the PG. Coexpression of *UAS-p35* and *UASp-TnBVank1* in the same PG cells with *phm-Gal4* driver did not rescue the developmental arrest phenotype (n = 58). Collectively, these data indicate that the developmental arrest induced by *TnBVank1* does not depend on the reduced PG size triggered by apoptosis, but on its capacity to disrupt PG functioning when expressed before the production of the 20E peak.

### *Tn*BVANK1 affects the cytoskeletal network in the PG cells

The altered *TnBVank1* PG cell morphology and the associated mislocalization of mCD8::GFP prompted us to analyze the cytoskeletal network in these cells.

**Figure 4:**
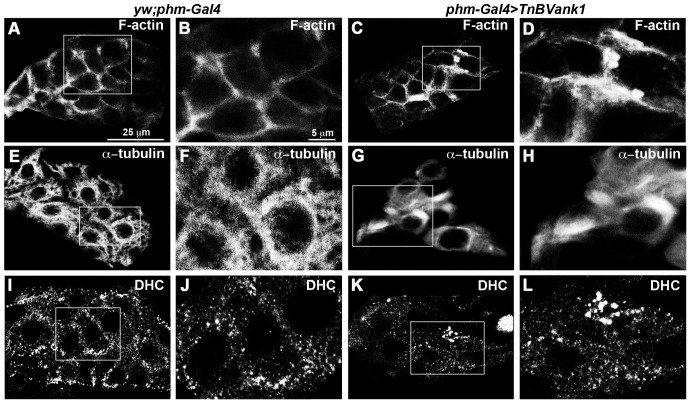
*TnBVank1* PG cells have an altered cytoskeleton. Phalloidin staining in control (A,B) and in *TnBVank1* (C,D) PG cells. F-actin shows an altered distribution, characterized by thick masses of filaments in *TnBVank1* PG cells. (E–H) α-tubulin-GFP fusion protein was expressed in *yw;phm-Gal4* and *TnBVank1* PG to investigate the microtubule network. Compared to control (E,F), in *TnBVank1* the microtubule cytoskeleton is strongly affected and forms bundles (G,H). (I–L) Immunostaining with anti-Dynein heavy chain shows that, compared to control (I,J), in *TnBVank1* PG cells the cortical localization of this protein is reduced and characterized by an evident dotted distribution (K,L). For each immunostaining we analyzed 60 PGs of five days AED larvae. PGs in panels A,C,E,G,I,K are at the same magnification and the reference scale bar is 25 µm and showed in A. Boxed regions are magnified in B,D,F,H,J,I and the reference scale bar 5 µm is indicated in B.

We investigated F-actin and α-tubulin distribution in *TnBVank1* PGs and we observed an altered cytoskeletal organization in all analyzed glands (n = 60). As shown by phalloidin staining (Figure 4A–D), cortical actin did not appear regularly distributed in *TnBVank1* PG cells, in which thick masses of actin filaments were detected (Figure 4C,D). The microtubule network was investigated by analyzing the distribution of α-tubulin-GFP fusion protein, which was coexpressed with *TnBVank1* in the PG. Compared to control, expressing only α-tubulin-GFP protein (Figure 4E,F), the cytoskeleton of the *TnBVank1* PG cells appeared strongly affected, as shown by the formation of thick bundles of microtubules (Figure 4G,H). The dynamic function of the microtubule network was then analyzed in *TnBVank1* PGs (n = 60) by assessing the distribution of the minus-end-directed microtubule motor dynein, using an anti-Dynein heavy chain antibody [20]. Compared to the control (Figure 4I,J), cells of *TnBVank1* PG displayed a reduced cortical distribution of dynein, along with some large dynein dots (Figure 4K,L). These data indicate that the whole cytoskeletal network is markedly altered in the PG cells expressing *Tn*BVANK1. We also analyzed *Gal80^ts^-TnBVank1* PG cells at different time points (96 h AED, 72 h AED and 48 h AED) and we observed that F-actin organization is strongly altered when larvae were shifted to restrictive temperature at 48 h AED. This suggests that the prolonged expression of *TnBVank1* during development is causative of the disruption of cytoskeleton (Figure S1). Moreover, as discussed above, no adult phenotypic effect or developmental delay was produced by the expression of *TnBVank1* in different tissues, using a wide range of tissue specific *Gal4* drivers (Table S1). This suggests that the cytoskeletal structure is not affected in all tissues, as can be observed in the fat body (Figure S2). This is further corroborated by our previous study showing that in the *Drosophila* oocyte *Tn*BVANK1 interferes with proper microtubule and microtubule-motor protein functions [16], and does not affect the overall cytoskeletal structure.

### *Tn*BVANK1 expression alters the cholesterol trafficking endocytic pathway of PG cells

**Figure 5:**
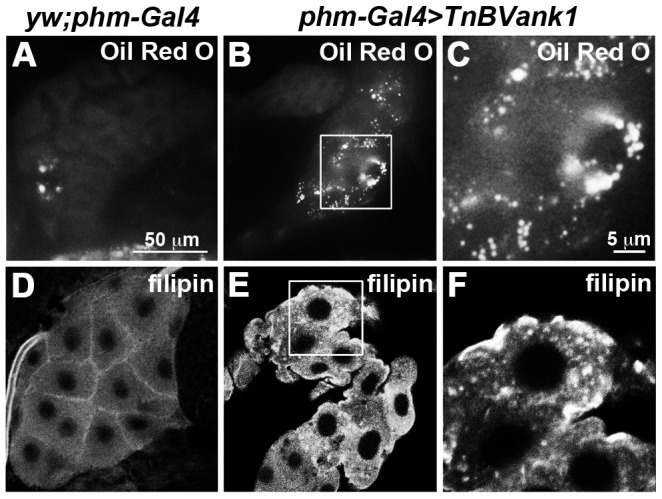
*TnBVank1* PG cells show lipids accumulation. (A) In the control *yw;phm-Gal4* there are few lipid droplets stained with Oil Red O, while in *TnBVank1* cells several lipid droplets are detected (B,C). (E,F) In *TnBVank1* there is also a sterol accumulation, shown by filipin staining, which is absent in control PG (D). 60 PGs were stained for each experiment. PGs in panels A,B,D,E are at the same magnification and the reference scale bar 50 µm is showed in A. Boxed regions are magnified in C,F and the reference scale bar is 5 µm indicated in C.

The observed negative impact of *Tn*BVANK1 on the cytoskeleton of PG cells may reduce the level of ecdysteroid biosynthesis by disrupting the uptake, transport and trafficking of sterols, essential steps for ecdysteroid biosynthesis [34]. Cholesterol, which cannot be synthesized by insects [35], enters the steroidogenic cells through a receptor-mediated low-density lipoprotein (LDL) endocytic pathway [36], which targets cholesterol to the endosomes. It is then transformed into 7-dehydrocholesterol in endoplasmic reticulum and transported to other subcellular compartments through further metabolic steps of the ecdysteroidogenic pathway [35]. We analyzed lipid vesicular internalization and trafficking in the *TnBVank1* PG cells with a staining procedure using Oil Red O. Conversely to control (Figure 5A), in all *TnBVank1* PGs analyzed (n = 60), we observed a varying level of evident increased accumulation of lipid droplets (Figure 5B,C). Then, using filipin, which specifically stains non-esterified sterols [37], compared to control (Figure 5D), *TnBVank1* PGs (n = 60) showed a marked cholesterol accumulation in discrete vesicular drops (Figure 5E,F). These data suggest that *Tn*BVANK1 does not affect lipid uptake, but that the endocytic pathway is in some way disrupted.

**Figure 6:**
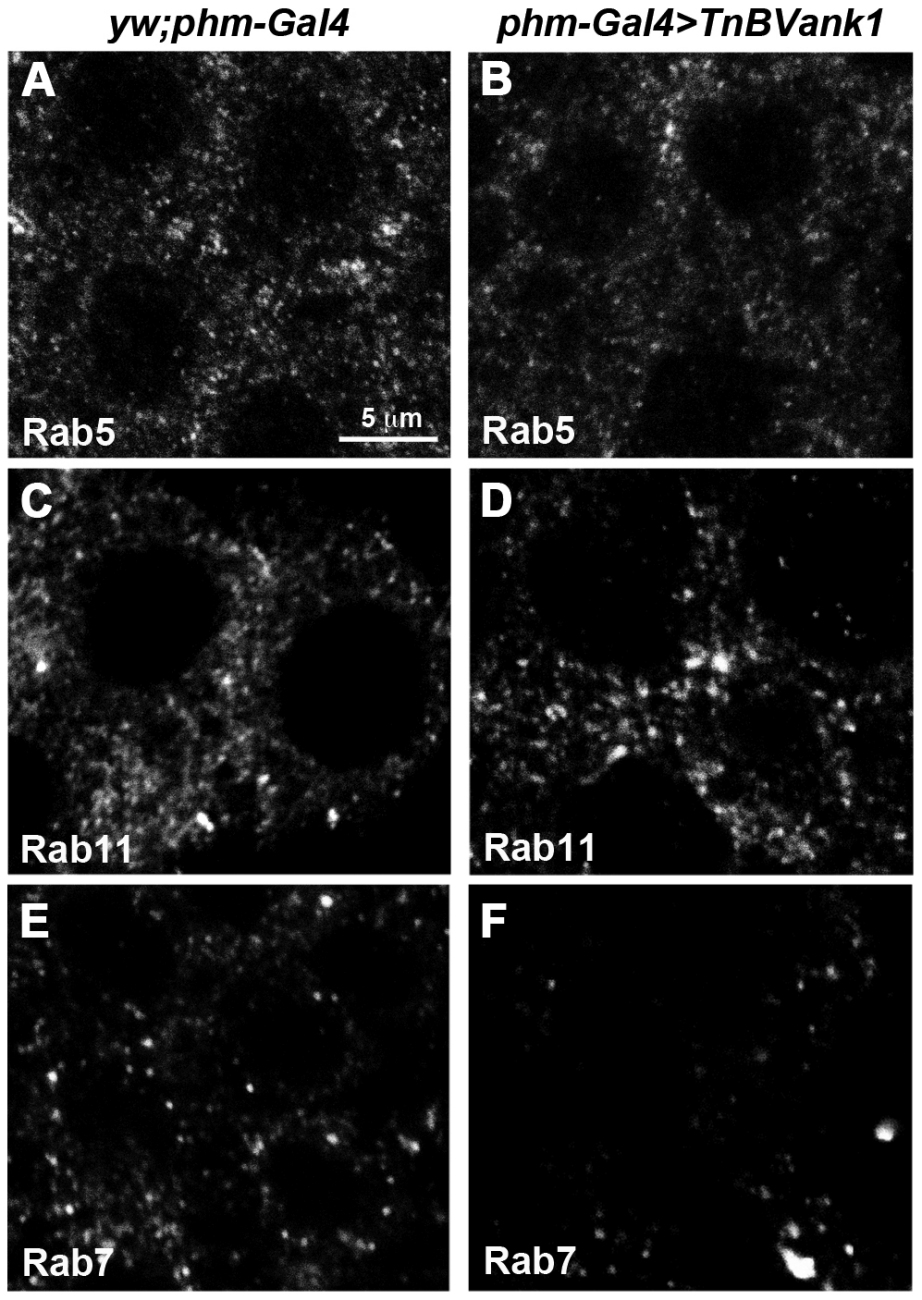
*Tn*BVANK1 disrupts the endocytic pathway in PG cells. 60 PGs stained for Rab5 (A,B), Rab11 (C,D) and Rab7 (E,F) in *yw;phm-Gal4* and *TnBVank1* larvae at five days AED. The distribution of endosomes marked with Rab5 (A,B) and Rab11 (C,D) is not affected by *TnBVank1* expression, while a reduction in number was observed for late endosomes marked with Rab7 (E,F). All panels are at the same magnification and the reference scale bar is 5 µm showed in A.

The endocytic pathway is organized into three major compartments, each characterized by specific Rab GTPase proteins that can be used as tags for the different endosomes [38]. Early endosomes are enriched in Rab5, late endosomes are associated with Rab7, and Rab11 marks the recycling endosomes. We used antibodies directed against these Rab proteins to investigate the endocytic pathway in PG cells (n = 60 PGs for each experiment) [19]. The cellular distribution of the early (Figure 6A,B) and recycling endosomes (Figure 6C,D) appeared to be comparable between PGs of control (Figure 6A,C) and of *TnBVank1* larvae (Figure 6B,D). Whereas, compared to control (Figure 6E), in *TnBVank1* PGs few Rab7 positive vesicles were detected (Figure 6F). This suggests that *Tn*BVANK1 may somehow affect the endocytic pathway.

**Figure 7:**
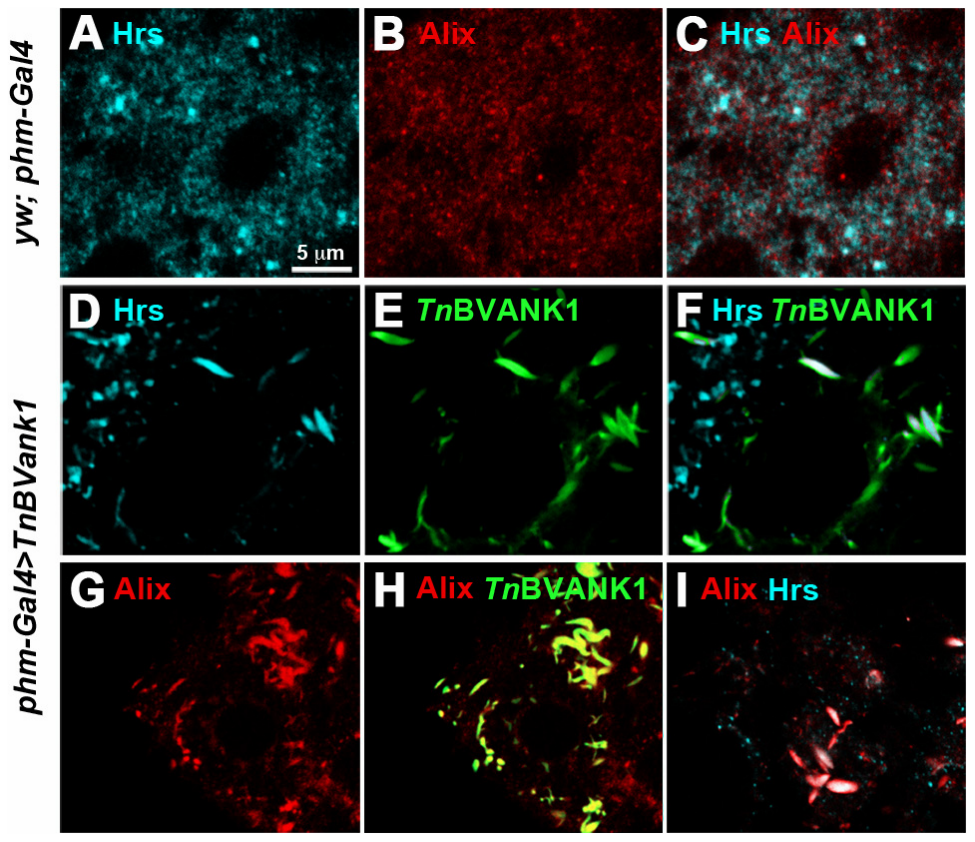
*Tn*BVANK1 protein colocalizes with Hrs- and Alix-positive vesicles. Confocal images of PG of *yw;phm-Gal4* (A–C) and *TnBVank1* (D–I) larvae stained for Hrs (cyan), Alix (red) and *Tn*BVANK1 (green). In the control cells Alix (B) and Hrs (A) are widely distributed in the cytoplasm and their signals partially overlap (C). In *TnBVank1* cells (D,F) a number of vesicles marked by Hrs have different shape compared to those present in controls (A,C). These modified vesicles show a strong colocalization with *Tn*BVANK1 signal (E,F), demonstrating that *Tn*BVANK1 protein is associated with Hrs-marked vesicles. In *TnBVank1* cells (G) most of Alix-marked vesicles have different shape compared to those present in controls (B). Immunostaining with anti-Alix and anti-*Tn*BVANK1 shows a strong colocalization of *Tn*BVANK1 and Alix signals in the stroke-shaped vesicles (H). In these modified vesicles the Alix and the Hrs signals are both detected (I). PGs in all panels are at the same magnification and the reference scale bar is 5 µm indicated in A.

We then analyzed the PG distribution of endosomes carrying the Hepatocyte growth factor-regulated tyrosine substrate (Hrs) (Figure 7A). This protein regulates inward budding of endosome membrane and multivesicular bodies (MVBs)/late endosome formation [22]. Interestingly, quite a few Hrs marked vesicles in the *TnBVank1* PG cells showed the stroke-shaped form associated with *Tn*BVANK1 signals (Figure 7D,E). In addition, most of the immunodetection signals of *Tn*BVANK1 (Figure 7E) colocalized with the Hrs marked vesicles (Figure 7F; Pearson's coefficient = 0.96±0.06). In contrast, most of these vesicles showing a normal round shape did not colocalize with *Tn*BVANK1. This finding suggests an interaction of *Tn*BVANK1 with endosome associated proteins, which may partly account for the observed alterations of the endocytic trafficking routes.

MVBs formation is controlled by a set of proteins, the endosomal sorting complex required for transport, ESCRT-0 to III, which sequentially associate on the cytosolic surface of endosomes [39]. A partner of the ESCRT proteins, which also regulates the making of MVBs, is the ALG-2-interacting protein X (Alix), first characterized as an interactor of apoptosis-linked gene protein 2 (ALG-2) [40]. It has been reported that the late endosomal lipid lysobisphosphatidic acid (LBPA) and its partner protein Alix play a direct role in cholesterol export [41]. Therefore, by using an antibody directed against Alix, we analyzed the distribution of this protein in control and *TnBVank1* PGs (Figure 7B,G). According to its multifunctional activity [42], Alix was found widely distributed in the cytoplasm of wild type cells (Figure 7B), and, as expected, marked some Hrs positive vesicles (Figure 7C). Interestingly, in the *TnBVank1* PG cells the *Tn*BVANK1 positive stroke-shaped structures showed a strong colocalization with Alix (Figure 7H; Pearson's coefficient = 0.99±0.07). In addition, several of these Alix positive stroke-shaped structures colocalized with Hrs (Figure 7I; Pearson's coefficient = 0.95±0.16), indicating that these are modified endocytic vesicles. This strong interaction of *Tn*BVANK1 with Alix containing vesicles and the altered cholesterol distribution observed in PG are concurrent evidences indicating that the cholesterol route was altered. Therefore, the interaction between *Tn*BVANK1 and endosomes specifically affects the endosomal trafficking of sterols, likely limiting their supply to subcellular compartments where ecdysteroid biosynthesis takes place [35].

## Discussion

PDVs are among the major host regulation factors used by parasitic wasps to subdue their hosts, which show immunosuppression and a number of developmental and reproductive alterations associated with disruption of their endocrine balance [3], [4], [6]. Relatively more studies have addressed the host immunosuppression mechanisms, focusing on virulence factors in the *ank* gene family largely shared among different taxa [43]. While an immunosuppressive function has demonstrated for the PDV *ank* gene family, if and how these viral genes impact endocrine pathways or other targets has not yet been addressed [11]. Here we report experimental evidence demonstrating the role of a *Tn*BV *ank* gene in the disruption of E biosynthesis and the induction of developmental arrest.

The proteins encoded by PDV *ank* genes show significant sequence similarity with members of the IκB protein family involved in the control of NF-κB signaling pathways in insects and vertebrates [44]. Because they lack the N- and C-terminal domains controlling their signal-induced and basal degradation, they are able to bind NF-κB and prevent its entry into the nucleus to activate the transcription of genes under κB promoters [15], [45], [46]. The *ank* gene family is one of the most widely distributed in PDVs and contains members which are rather conserved across viral isolates associated with different wasp species [10], [15], [46]–[48]. These genes likely originate from horizontal gene transfer from a eukaryote, which could be the wasp, the host or another organism. Indeed, the nudiviruses, ancestors of bracoviruses [7], do not encode any gene showing similarity with *ank* family members. Their multiple acquisition and stabilization in different evolutionary lineages are clearly indicative of the key role they play in successful parasitism. This also suggests that *ank* genes may be involved in multiple tasks on host parasitization, by influencing different physiological pathways.

**Figure 8:**
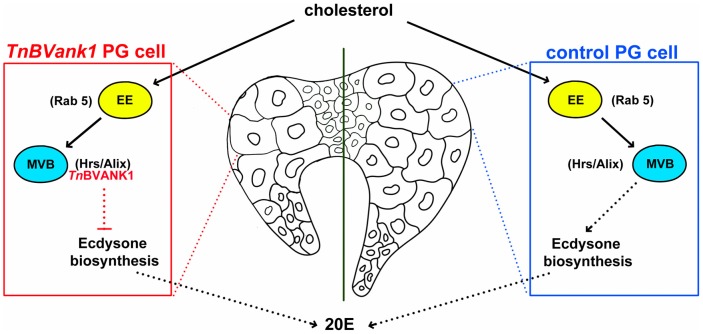
Schematic overview showing the step in which *Tn*BVANK1 affects the ecdysone biosynthesis in the PG cells. EE: early endosomes, MVB: multivesicular bodies.

Here, we provide experimental data that corroborate this hypothesis for *TnBVank1,* a gene of the bracovirus associated with the wasp *T. nigriceps* (*Tn*BV), which parasitizes the larval stages of the tobacco budworm, *H. virescens*. Using *Drosophila* as a model system, we show that the *Tn*BVANK1 protein acts as a virulence factor disrupting E biosynthesis (Figure 8) and causes developmental arrest of the larvae, which fail to pupariate. The number of late endosomes is reduced in the *TnBVank1* expressing cells and this is concurrent with an interesting change of Hrs-*Tn*BVANK1 positive vesicle morphology. This defective mechanism in MVB and late endosome formation is accompanied by an evident alteration of sterol trafficking as indicated by the accumulation of lipid and sterol-rich vesicles.

Cholesterol is processed to free cholesterol by lipase in the endosomal compartment and after that it moves to other compartments entering the ecdysone biosynthesis machinery [34]. Recent evidences from mammalian cell studies indicate that the late endosomal lipid LBPA and its partner Alix play a role in controlling the cholesterol export from endosomes [41]. Our finding that *Tn*BVANK1 interacts with Alix positive vesicles and affects the sterol delivery suggests that Alix function in cholesterol export is conserved between *Drosophila* and mammals.

Our data let us to hypothesize that in *TnBVank1* expressing PG cells cholesterol may be trapped into the MVBs. This block leads to insufficient sterol supply to reach the ecdysone level necessary to complete development. Interestingly, the fact that *TnBVank1* expression in other tissues did not alter development suggests that *Tn*BVANK1 impact on cholesterol trafficking may deeply affect the PG cells engaged in an intense steroidogenic activity.

We show that *Tn*BVANK1 disrupts the cytoskeletal structure of PG cells, and this appears to be a PG specific alteration. Indeed, in our previous work we demonstrated that the targeted expression of this *ank* gene in *Drosophila* germ cells alters microtubule network function in the oocyte, as shown by the mislocalization of several maternal clues, without affecting the cytoskeletal structure [16]. Therefore, we cannot exclude that the specific targeted effect of *Tn*BVANK1 on the cytoskeleton function of PG cells may have a negative impact on ecdysteroidogenesis. However, it can also be true that the disruption of the cytoskeletal structure of these cells could be a downstream consequence of the impaired steroidogenic activity. The altered cell physiology and the consequent accumulation of lipids and sterols may have wide-ranging and more generalized effects on cell architecture/dynamics and survival. In fact, the prolonged expression of *TnBVank1* by *phm-Gal4* during larval development causes cytoskeleton alteration and also apoptosis of a few cells, which may partly account for the observed reduction of the PG size.

It is interesting to note that the developmental arrest at L3 larval stage induced by *TnBVank1* expression in the PG perfectly mimics the developmental alteration of parasitized tobacco budworm larvae, which can regularly undergo larval molting but ultimately fail to pupate [13], [49]. The reduced gland size observed in parasitized larvae and the low basal production of ecdysteroids [14], [50] are fully compatible with a general reduction of the biosynthetic activity likely induced by *ank* genes. However, in naturally parasitized larvae these symptoms are also associated with a disruption of PTTH signaling, which requires active *Tn*BV infection of PG, where different viral genes are expressed [13], [51]. The high similarity of the recorded phenotypes represents a solid background on which to design specific experiments on the natural host. Indeed, the results reported here set the stage for specific *in vivo* studies in parasitized host larvae, that will have to address the respective roles of different *Tn*BV genes in the suppression of ecdysteroidogenesis.

## Supporting Information

Figure S1 **Prolonged expression of** ***TnBVank1*** **in PG cells during development alters cytoskeleton structure.** Phalloidin staining in PGs from *Gal80^ts^-phm-Gal4* and *Gal80^ts^-TnBVank1* larvae raised at 21°C (cyan) for different time intervals, then shifted at 31°C (red) and dissected at 120 h AED. PG cell cytoskeleton from *Gal80^ts^-TnBVank1* larvae incubated at 21°C until 96 h AED (D) or until 72 h AED (E) shows no significant differences from *Gal80^ts^-phm-Gal4* (A,B). F-actin cytoskeleton is completely altered in PG cells of *Gal80^ts^-TnBVank1* larvae incubated at 21°C until 48 h AED (F) compared to the control treated in the same condition (C). PG cells in all panels are at the same magnification and the reference scale bar 5 µm is indicated in A.
(TIF)

Figure S2 **Expression of** ***TnBVank1*** **in fat bodies does not affect cell morphology.** Phalloidin staining in fat bodies from the control *yw; lsp2-Gal4; UAS-mCD8::GFP* (A,B) and from fat bodies expressing *TnBVank1 lsp2-Gal4, UAS-mCD8::GFP/TnBVank1* (C,D). Fat bodies are at the same magnification in all panels and the scale bar is indicated in A.
(TIF)

Table S1 **Effects of** ***TnBVank1*** **expression using different** ***Gal4*** **drivers.**
(PDF)
