# Supplementary figures and images for: A Polydnavirus ANK Protein Acts as Virulence Factor by Disrupting the Function of Prothoracic Gland Steroidogenic Cells
Source: PLoS One. 2014 Apr 17;9(4):e95104. doi: 10.1371/journal.pone.0095104 (PMC3990622; doi:10.1371/journal.pone.0095104)

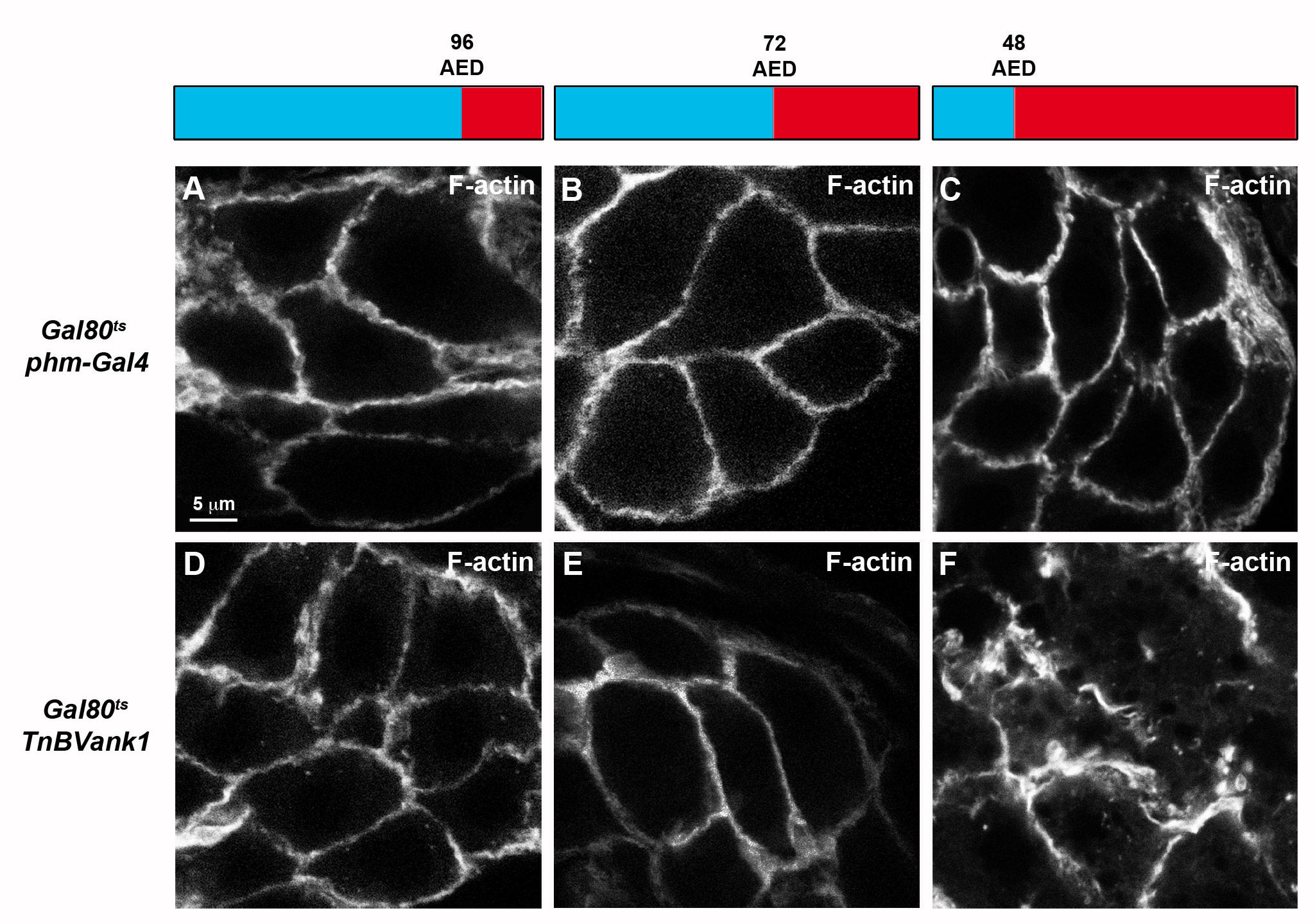

Supplement: Figure S1 — Prolonged expression of TnBVank1 in PG cells during development alters cytoskeleton structure. Phalloidin staining in PGs from Gal80ts-phm-Gal4 and Gal80ts-TnBVank1 larvae raised at 21°C (cyan) for different time intervals, then shifted at 31°C (red) and dissected at 120 h AED. PG cell cytoskeleton from Gal80ts-TnBVank1 larvae incubated at 21°C until 96 h AED (D) or until 72 h AED (E) shows no significant differences from Gal80ts-phm-Gal4 (A,B). F-actin cytoskeleton is completely altered in PG cells of Gal80ts-TnBVank1 larvae incubated at 21°C until 48 h AED (F) compared to the control treated in the same condition (C). PG cells in all panels are at the same magnification and the reference scale bar 5 µm is indicated in A. (TIF) [file pone.0095104.s001.tif]

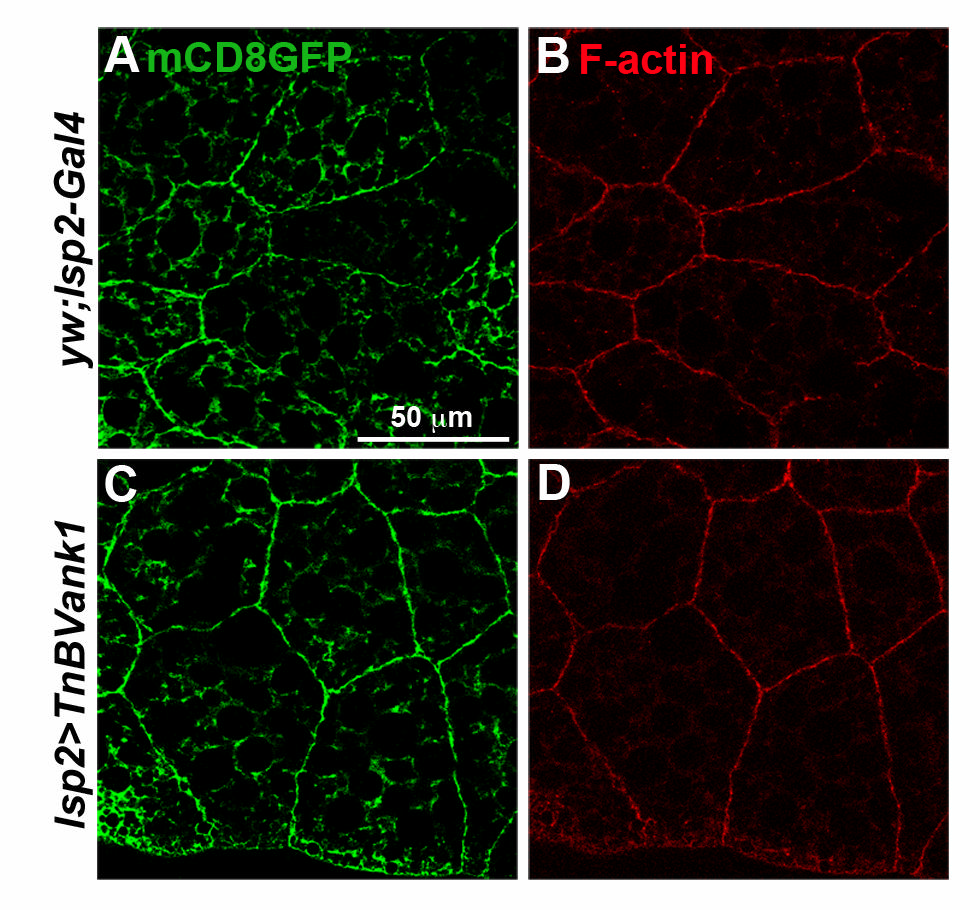

Supplement: Figure S2 — Expression of TnBVank1 in fat bodies does not affect cell morphology. Phalloidin staining in fat bodies from the control yw; lsp2-Gal4; UAS-mCD8::GFP (A,B) and from fat bodies expressing TnBVank1 lsp2-Gal4, UAS-mCD8::GFP/TnBVank1 (C,D). Fat bodies are at the same magnification in all panels and the scale bar is indicated in A. (TIF) [file pone.0095104.s002.tif]
