# Supplementary material for: A Polydnavirus ANK Protein Acts as Virulence Factor by Disrupting the Function of Prothoracic Gland Steroidogenic Cells
Source: PLoS One. 2014 Apr 17;9(4):e95104. doi: 10.1371/journal.pone.0095104 (PMC3990622; doi:10.1371/journal.pone.0095104)
Supplement: Table S1 — Effects of TnBVank1 expression using different Gal4 drivers. (PDF) [file pone.0095104.s003.pdf]

Table S1. Different *Gal4* drivers were used to target expression of *TnBVank1* to specific tissues.

Gal4 driver (Bloomington stocks)	Tissue specificity	Effects on developmental timing	% of normal adults (*)
Eyeless (#5535)	eye disc	None	100 (n=95)
Scabrous (#6479)	eye disc, bristles, proneural clusters, sensory organ precursor cells	None	100 (n=89)
Appl (#32040)	larval nervous system	None	100 (n=66)
Hemolectin (#30140)	lymph glands, circulating hemocytes	None	100 (n=58)
Elav (#8760)	nervous sysytem	None	100 (n=88)
Lsp2 (#6357)	third instar fat body	None	100 (n=80)
Hemese (#8700)	hemocytes and salivary glands	None	100 (n=83)
337Y (#32119)	all late embrionic tissues	None	100 (n=112)
T80 (#1878)	ubiquitous in third instar imaginal discs	None	100 (n=81)
c885a (#6990)	larval optic lobes, wing discs, fat body, leg and eye disc peripodial membranes	None	100 (n=65)

(*) For each cross the percentage (%) of normal adults is calculated by dividing the number of normal adults with total number of the same genotype animals.
